# Photic Retinopathy: Diagnosis and Management of This Phototoxic Maculopathy

**DOI:** 10.3390/life15040639

**Published:** 2025-04-12

**Authors:** Mihaela Madalina Timofte Zorila, Livio Vitiello, Filippo Lixi, Alessia Coppola, Feyza Cukurova, Alfonso Pellegrino, Giuseppe Giannaccare

**Affiliations:** 1 Faculty of Medicine, “Grigore T. Popa” University of Medicine and Pharmacy, 16 Universitatii Street, 700115 Iasi, Romania; mihaela-madalina.timofte-zorila@d.umfiasi.ro; 2 Department of Ophthalmology, Cai Ferate Clinical Hospital, 1 Garabet Ibraileanu Street, 700506 Iasi, Romania; 3 Eye Unit, “Luigi Curto” Hospital, Azienda Sanitaria Locale Salerno, 84035 Polla, Italy; alessiacoppola330@gmail.com (A.C.); al.pellegrino@aslsalerno.it (A.P.); 4 Eye Clinic, Department of Surgical Sciences, University of Cagliari, 09124 Cagliari, Italy; f.lixi0106@gmail.com (F.L.); giuseppe.giannaccare@gmail.com (G.G.); 5 Department of Ophthalmology, Mardin Training and Research Hospital, 47100 Mardin, Turkey; feyzacukurova143@gmail.com

**Keywords:** arc welding maculopathy, handheld laser maculopathy, iatrogenic macular degeneration, photic retinopathy, solar maculopathy

## Abstract

Photic retinopathy is an uncommon clinical entity characterized by retinal damage brought on by excessive exposure to light without protection. It encompasses several distinct clinical categories, including solar maculopathy, handheld laser maculopathy, arc welding maculopathy, and iatrogenic macular degeneration. These clinical entities result from exposure to diverse light sources, such as solar radiation, laser pointers, welding arcs, and operating microscopes during ophthalmic procedures. Patients typically present with bilateral but asymmetric symptoms, including reduced visual acuity, central or paracentral scotomas, photophobia, metamorphopsia, and headaches. After exposure, most people can recover on their own in a matter of weeks to six months without the need for special care. However, thanks to their anti-inflammatory properties, several clinical cases reporting the use of steroids for acute photic retinopathy have been documented in the scientific literature, together with the use of antioxidants. The purpose of this review is to provide an update on this phototoxic maculopathy, describing its different clinical entities, diagnosis, and treatment options, and also focusing on the role of optical coherence tomography for its management.

## 1. Introduction

Photic retinopathy or retinal phototoxicity is a retinal condition resulting from excessive exposure to intense light sources [1], for variable periods of time, causing possible permanent visual injury, particularly affecting the central vision [2]. Despite the fact that direct gazing at the sun or a solar eclipse without the necessary protection is the most recognized cause, other factors such as welding, unprotected sunbathing, laser pointer exposure [3,4,5], and even reflections from surfaces including water, sand, or snow have also been noticed in the past years [6,7,8,9]. With the increased use of leading technology, cases also emerge after the exposure to laser-induced plasma flashes during surgical procedures, xenon arc exposure, or flying without protective goggles [10,11,12,13,14].

The pathophysiology of photic retinopathy is a complex and intricate process involving various mechanisms, including photothermal and photochemical injury of the retina [1]. The exact mechanisms behind photic retinopathy are not entirely understood, but several hypotheses have been brought forward to explain the noticed pathophysiological changes. One of the leading hypotheses regarding the pathophysiology of photic retinopathy involves the production of a toxic photoproduct derived from vitamin A at the time of exposure to intense light [15]. This photoproduct is presumed to have a toxic effect on the retinal pigment epithelium (RPE), leading to the development of characteristic alterations observed in photic retinopathy [16].

Optical coherence tomography (OCT) has significantly enriched our understanding of the retina’s structural changes in photic retinopathy cases [17]. It has become valuable in diagnosing and managing this retinal disease by documenting structural changes, including modifications in the RPE, photoreceptor outer segments, and the outer nuclear layer [15,18]. OCT provides high-resolution sectional images of the retina, enabling detailed observation of the modifications following photic injury, identifying disruptions within the retinal layers, and monitoring the capacity for visual recovery or further deterioration [19].

In this review, we underline the different clinical entities of macular phototrauma and the role of OCT in understanding the etiology and management of several types of retinal injuries that fall under the shade of photic retinopathy. We discuss how OCT has revolutionized the diagnosis of the aforesaid ocular diseases, highlighting its ability to detect early alterations in the macula and assess the functional and structural outcomes of retinal injury, in addition to current potential available treatments for photic retinopathy. Clinicians have to be aware of the potential risk factors involving multiple causes of photic retinopathy and its distinctive OCT findings, as early awareness and appropriate management can help prevent permanent visual disability in affected patients.

## 2. Materials and Methods

**Figure 1:**
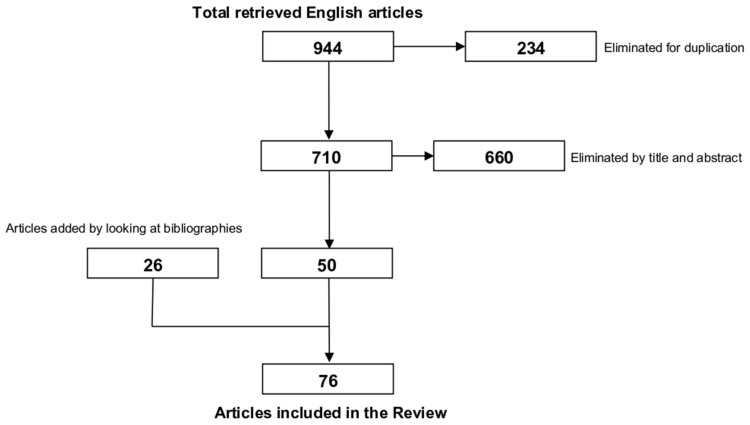
Graphical scheme of the bibliographic search.

A comprehensive literature search was performed on Google Scholar, PubMed, ScienceDirect, and Scopus databases using the following search terms: “(macular phototrauma) OR (photic maculopathy) OR (solar maculopathy) OR (eclipse maculopathy) OR (phototoxic maculopathy)”. After searching the specified databases, we found 944 articles, of which 234 were identified as duplicates and removed. All abstracts (710 articles) were carefully screened, and relevant articles were selected for inclusion based on their correlation to the topic. Of these, only 50 papers were included according to our proposed criteria. In order to locate any other articles that could be relevant to the current research, we additionally and manually examined the reference lists of the included studies in this review (Figure 1).

The search was carried out in January 2025 and the inclusion criteria were studies documenting photic retinal injury published between 1990 and 2025, exclusively in English, which included case reports, case series, original research articles, and reviews. Moreover, only studies that used OCT as part of the diagnostic process for this clinical entity were considered, while studies involving multiple retinal pathologies were excluded from the review to ensure a focused evaluation of photic retinopathy.

## 3. Different Clinical Entities of Photic Retinopathy

Patients with photic retinopathy may complain sudden-onset central visual disturbances, including blurred vision, visual distortions [2], or central scotomas. The fovea, the unit responsible for sharp central vision, is particularly vulnerable to light-induced impairment due to its high concentration of photoreceptor cells. These modifications are revealed by electron microscopy, proving significant swelling of photoreceptor outer segments, with membrane separation and degeneration of lamellar disks. A granular substance with irregular density was present between the outer segments and the RPE. Inner segment mitochondria were enlarged, and some photoreceptor and Müller cells showed cytoplasmic breakdown [20].

Solar retinopathy or eclipse retinopathy develops after direct exposure to sunlight, especially during solar eclipses without proper eye protection. The injury results in a complex condition influenced by multiple factors, including the characteristics of light exposure, individual susceptibility (such as ocular pigmentation, refractive error, and age), and environmental factors [17]. Intensity and duration of light exposure are meaningful predictors of retinal damage. Photoreceptors can be damaged by radiation with wavelengths from 400 to 1400 mM, while the cornea and lens absorb wavelengths between 10 and 400 nm [18]. Yannuzzi et al. displayed the influence of ocular media clarity and solar retinopathy development [21]. Young, healthy individuals, particularly young men, emmetropes, and those with mild hypermetropia, are at higher risk because the light is more directed towards the retina. Since intraocular lenses utilized for cataract surgery typically include ultraviolet (UV) protection [22], the risk of solar retinopathy is exacerbated in aphakic eyes. On the other hand, the formation of cataract can provide some protection against light exposure and solar retinopathy development [17]. The size of the pupil leverages the amount of light that enters the eye. Dilated pupils, which permit more light to reach the retina, are associated with a higher risk of retinal damage. Conditions or activities that cause mydriasis, such as anxiety, physical exercise, or certain medications, may therefore predispose individuals to solar retinopathy injury [23]. Environmental conditions have an important role in the development of this ocular disease. In fact, high-altitude areas, where UV-B radiation is more intense due to lean atmospheric layers, are associated with an increased risk of solar retinopathy [24]. Likewise, highly reflective surfaces such as water, snow, sand, and glass may increase exposure to hazardous light by reflecting it into the eyes [21]. Snow is probably going to generate indirect solar retinopathy considering that it reflects 90% of the incident sunlight [25].

Laser-induced maculopathy is a retinal injury determined by exposure to high-power lasers in the majority of cases of retinal phototoxic injury (85.1%), while low-power lasers were less frequently associated. The shorter wavelengths of green (45.5%) and blue lasers (42.0%) were most commonly employed, compared to the longer wavelengths of red (11.6%), infrared (0.9%), and purple (0.9%) lasers [26]. Visible and near-infrared lasers (400–1400 nm) generate heat that clots proteins and disrupts retinal structures. Short, high-energy pulses can create shock waves, causing mechanical retinal rupture, while persistent exposure to lower intensities may generate oxidative stress and photoreceptor apoptosis [3,5]. Food and Drug Administration Class II or IIIa lasers with outputs of a maximum 5 mW and wavelengths between 632.8 and 670.0 nm are the laser pointers that are most frequently used as tools in platform presentations or as toys for teenagers [27]. With brief exposure durations and ocular defense systems like the blink reflex, these lasers are generally safe for human eyes. However, because the risk of injury varies depending on the laser’s wavelength, pulse duration, spot size, and irradiance, misuse of even low-power laser pointers may end up in retinal damage [5]. Lesions created by lasers have a rugged form, a distinctive dendritic structure, and linear streaks that extend outward. All clinicians should be aware of the potential for misdiagnosis with macular dystrophy in children [27].

Welding produces optical radiation with varying wavelengths and intensities, including visible, infrared, and UV light [8]. Welding arc retinopathy occurs due to exposure to arc welding without satisfactory eye protection, leading to acute thermal and photochemical retinal damage. The UV component (below 400 nm) induces photochemical damage by depositing reactive oxygen species (ROS), leading to lipid peroxidation, oxidative stress, and apoptosis in photoreceptors and the RPE [28]. Persistent or repeated exposure to intense visible and infrared light can cause thermal coagulation of retinal tissues, similar to laser blazes. Clinically, photo-keratitis, sometimes called “welder’s eye”, is the most frequent ocular damage presented as a foreign body feeling, discomfort, and impaired visual acuity typically appearing 6–12 h after exposure [8]. Welding arc retinopathy is associated with blurred vision, photophobia, and central or paracentral scotomas, with fundus examination sometimes revealing nuanced foveal changes. OCT can show outer retinal disruption, while fluorescein angiography can highlight areas of RPE damage. Unlike hand-held laser maculopathy, the outer retinal streak in this welding maculopathy instance does not show hypo-autofluorescence, while having a similar look on spectral domain OCT to laser-induced maculopathy [29]. Some authors suggested that autofluorescence observations in direct laser injury cases may be due to increased thermal energy exposure and concentration, resulting in higher retinal damage [9,29]. Prevention focuses on using protective welding helmets with appropriate filters to block harmful wavelengths and limiting direct exposure to welding arcs to prevent cumulative retinal injury.

Prolonged ophthalmic examination and exposure to operating microscopes can also lead to photochemical and thermal retinal damage. Light-emitting diode (LED) lighting systems have been the subject of numerous investigations, which have revealed a particularly high-risk potential at wavelengths below 500 nm [30,31,32]. There is also a significant risk associated with xenon lamps of similar irradiation [33]. Clinically, patients present with acute-onset central scotoma, reduced visual acuity, metamorphopsia, and photophobia, typically developing within hours of exposure. In cases of prolonged or high-intensity exposure, persistent central visual impairment may occur due to irreversible photoreceptor damage. Fundoscopic examination may reveal a subtle yellowish foveal spot, loss of the foveal reflex, or mild outer retinal whitening, indicative of early phototoxic injury. High-intensity light from surgical microscopes, especially in prolonged procedures like cataracts [34,35,36,37,38,39] or vitreoretinal surgery [40,41,42], can cause photochemical and thermal retinal damage, particularly in the macula, where light is focused. The mechanism involves cumulative oxidative stress, leading to ROS production, mitochondrial dysfunction, and photoreceptor apoptosis, resembling the photochemical injury seen in laser damage. The prevalence of retinal phototrauma during cataract surgery varies according to the surgical technique (extracapsular or intracapsular) and is related to the type of the study. In fact, in the retrospective studies the prevalence is estimated at 7%, while in prospective studies the reported percentage raises up to 28%. The risk of phototoxicity also increases along with surgery time: Byrnes et al. reported a risk of 5% at 10 min, reaching 80% at 90 min of preimplantation exposure [35]. In order to prevent phototoxic maculopathy at the vitreoretinal surgery point, it is recommended to maintain distance between the retina and the endoilluminator source [41]. There are also several studies documenting retinal lesions following femtosecond laser surgery [43,44,45].

## 4. Diagnosis

The majority of the patients report direct exposure to sunlight primarily from sungazing or observing a solar eclipse [1]. A thorough history helps to establish the exposure timeline and subsequent symptoms to determine the severity of the retinal damage. Rai et al. [46] reported in their study that only 51% of subjects affected by solar retinopathy referred to a sungazing history. These patients presented a diagnostic challenge due to the fact that various factors can lead to similar changes. However, the data gathered from imaging studies can aid in identifying the underlying cause of the impairment.

Advanced technologies in ophthalmic imaging have improved the capacity to diagnose and assess the severity of retinal injury. Fundus photography can be used as an early diagnostic tool. In the acute phase of solar retinopathy, it can unveil characteristic central foveal changes, a typical small yellow spot at the fovea, encircled by faint grey granular coloration. The yellowish discoloration will usually become faint with time, leaving a pathognomonic reddish spot, or it can return to near-normal aspect in the follow-up stages [17]. However, these changes are not always permanent and may vary depending on the extent of the damage and the time elapsed since exposure.

Regarding solar retinopathy, OCT is essential for identifying characteristic changes. While comparing fundus photography, which provides a surface view of the retina, OCT offers detailed cross-sectional, high-resolution, and three-dimensional images, allowing for more precise evaluation. The findings on OCT are highly time-sensitive, with distinct patterns emerging in the primary and late stages of solar retinopathy impairment. Aygün et al. enhanced the aspect of lesions in the acute phase (the first few days following exposure), where OCT may reveal focal areas of hyperreflectivity in the macula [47]. The outer retinal layers, including the outer segments of the photoreceptors and the RPE, are damaged, thus indicating photoreceptor inflammation [29]. Solar retinopathy affects Verhoeff’s membrane, which connects photoreceptors to the RPE [1], and a disruption of the external nuclear layer and the external limiting membrane is associated with poor prognosis.

At this stage, the changes on OCT are transitory, and the hyperreflectivity could resolve over time according to the healing process. This is why early OCT imaging is crucial to assess solar retinopathy, as it provides evidence of retinal impairment during the initial inflammatory response and also helps in tracking the recovery process and determining the reach of permanent damage [48].

**Figure 2:**
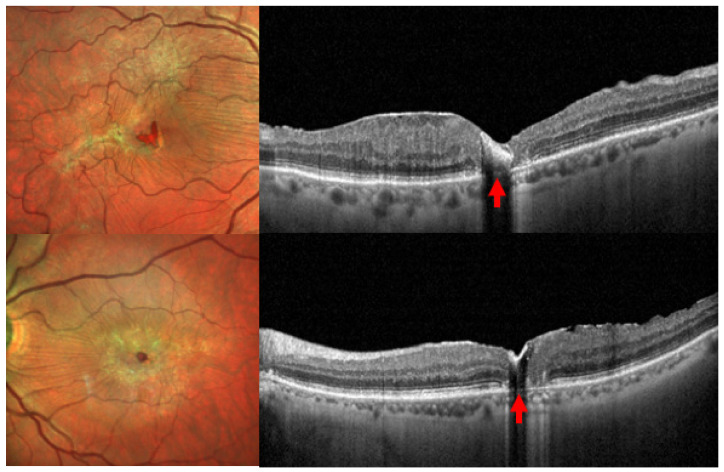
Fundus color photographs and optical coherence tomography scans of a bilateral chronic solar retinopathy. It is possible to detect the typical “outer retinal hole” (red arrows) located in the fovea, defined by a discontinuity of photoreceptor outer segments and the retinal pigment epithelium, which is characteristic of chronic solar retinopathy.

In chronic cases of solar retinopathy (weeks or even months after exposure), where extensive retinal injury has occurred, OCT reveals a characteristic strand of late solar retinopathy: an “outer retinal hole” located in the fovea. This is defined by a discontinuity in the outer retinal layers, specifically the photoreceptor outer segments and the RPE [49] (Figure 2).

The RPE injury is a sign of photochemical damage and these changes are frequently accompanied by choroidal abnormalities, which might include inflammation. Furthermore, the myoid and ellipsoid zones may exhibit focal disruption [50]. These findings are frequently linked with substantial visual impairment and may continue even in patients who experience complete visual recovery.

In laser-induced maculopathy, the acute phase is characterized by marked hyperreflectivity at the fovea, primarily involving the photoreceptor layer, RPE, and outer retina. This is frequently accompanied by ellipsoid zone and RPE disruption, indicative of direct photothermal or photomechanical damage [27]. In the chronic stage, persistent ellipsoid zone loss, focal atrophy of the outer retinal layers, and hyporeflective cavitations inside the RPE may be observed, signifying irreparable structural compromise [26]. Two non-pathognomic patterns have been identified for diagnostic purposes [27]. The presence of a focal rounded pattern is typical for lesions produced by third persons [51]; in contrast, the presence of retinal streaks is typical for self-inflicted lesions [29]. These streaks are generated by the Bell phenomenon: while gazing at a source of light for a long period of time, alterations having vertical orientation, located above the macula [29,52], secondary to exposure to laser pointers have been described and observed through OCT angiography [53].

Iatrogenic maculopathy, due to extended exposure to operating microscopes or examination phototoxicity, displays OCT changes ranging from subtle ellipsoid zone alterations in mild cases to pronounced outer retinal atrophy and cystoid spaces in severe cases [14]. These cystoid spaces may emulate parafoveal telangiectasia, entangling differential diagnosis [54,55]. Lesions are similar to other forms of photic retinopathy; the main differences are the extent of the lesion and the location above or below the fovea because the subjects do not focus into the light [54].

Similarly, welding arc retinopathy produces distinct OCT alterations: acute phase is very rare and often appears after 30 s of exposure [56]. Outer retinal hyperreflectivity is widespread, especially in the ellipsoid zone and RPE, which may indicate photothermal damage [8,18]. Chronic modifications over time include gradual outer retinal thinning, ellipsoid zone disruptions, and modest foveal atrophy, which are the result of mounting phototoxicity and cell death. In severe cases, chronic retinal changes can result in functional disability, emphasizing the importance of early detection and protective means in at-risk patients [9].

While OCT focuses on structural changes, it can also be tied in with functional testing (microperimetry or electroretinography) to assess the clinical impact of the retinal damage. This dual perspective helps to determine the functional bearing of the observed structural changes [1]. Microperimetry provides information regarding the functional impact by measuring retinal sensitivity and detecting areas of decreased light sensitivity, which frequently manifest as central or paracentral scotomas [57]. These functional changes may resolve over time, with scotomas decreasing in size or disappearing a few months after the exposure [58].

Multifocal electroretinography is a highly sensitive test used to appreciate the electrical responses of the retina, making it effective for identifying early and subtle macular dysfunction. In the solar retinopathy context, this diagnostic evaluation can uncover reduced amplitudes in both the fovea and parafovea. While these abnormalities often amend over time, some patients may still exhibit residual dysfunction, even after fully regaining their visual acuity [59,60].

Fundus autofluorescence is a non-invasive imaging method that utilizes the autofluorescent capacity of lipofuscin, which accumulates in the RPE. In cases of solar retinopathy, fundus autofluorescence typically unveils a well-defined hypoautofluorescent central area, which corresponds to the lack of photoreceptor cells due to RPE damage. This central area is encircled by an irregular halo of hyperautofluorescence, indicating the accumulation of outer retinal segments subsequent to photoreceptor injury [61].

## 5. Treatment

The majority of individuals with photic retinopathy report full vision recovery without special therapy within weeks to six months after exposure, and the visual prognosis is often excellent [62]. However, recovery outcomes exhibit considerable individual variation. Different factors influence this variability in clinical recovery, such as the type and the extension of the damage, in addition to the duration and the wavelength of the damaging light. Accordingly, the extension of retinal impairment may predict the severity of the damage, since an involvement of the inner retinal layers can lead to a permanent insult to the retinal tissue [63]. Similarly, the duration and the wavelength of the light may influence the damage severity, as the photochemical injury is associated with both long-duration exposure times as well as lower-wavelength light exposure [63]. Moreover, Atmaca et al. reported that the improvement in visual acuity was more prominent and earlier in eyes with a visual acuity of 0.2 or better at presentation [64].

Nonetheless, it was thought that antioxidants would offer a protective role in case of photic retinopathy since photochemical damage results in the generation of free radicals and oxygen-dependent toxicity to the outer retina. Vitamin C is considered a crucial antioxidant that may counteract superoxide radicals and shield the retina from harm caused by light [65]. However, it is uncertain if antioxidant supplementation in cases of photic retinopathy might improve visual recovery, even though antioxidants lower the risk of age-related macular degeneration development [66] and have some positive effects in animal models of phototoxicity. In fact, people affected by photic retinopathy generally heal completely on their own without any treatment.

Systemic corticosteroids have been utilized because of their strong anti-inflammatory properties [67], although there are currently no recommendations for their use in the treatment of acute photic retinopathy. Methylprednisolone is believed to reduce light-induced retinal damage by inhibiting lipid peroxidation [67]. For this reason, it has been used in a number of trials to treat photic retinopathy [68,69,70,71].

Although their findings first appear promising since they showed that macular edema has resolved, macular edema frequently goes away on its own, with or without corticosteroids [68,69,70,71]. Additionally, the ultimate visual acuity of individuals treated with systemic steroids ranged widely, from total remission to long-lasting impairment with central/paracentral scotomas [68,69,70,71]. Thus, the results of using systemic corticosteroids in patients with photic retinopathy have been inconsistent to date.

There is a wide range in the time between the initial light insult and the patient seeking for ophthalmic care. Nevertheless, the advantages of corticosteroids appear to be restricted to the initial period following exposure to light. For this reason, ophthalmologists should thus determine how long it has been since the initial exposure and should pay attention when administering systemic corticosteroids due to the possibility of systemic adverse effects, and the development of central serous chorioretinopathy [72].

## 6. Discussion

Photic retinopathy is often a self-limited condition that resolves completely in a matter of weeks to months. In rare cases, retinal damage can result in long-term vision loss and severe limitations in the patient’s ability to carry out everyday tasks, since destructive effects of UV light on the retina are possible. In fact, the posterior retinal pole, which is in charge of high visual acuity, is harmed by excessive UV radiation, with a small photothermal component and photochemical processes mostly responsible for this damage.

Individual response rates and recoveries vary because of the wide range of lesions and high individual heterogeneity. For this reason, the greatest preventative measure is definitely to avoid looking at the sun or watching solar eclipses, while lifeguards, ski instructors, laser engineers, welders, and others who operate in bright light conditions require appropriate protection.

Concerning the diagnosis, a comprehensive history together with multimodal imaging (OCT, fluorescein angiography, fundus autofluorescence, and multifocal electroretinography) allows clinicians to detect retinal photodamage early. In particular, the role of OCT is crucial for the diagnosis of photic retinopathy, since it permits to identify the characteristic signs of retinal damage for each different clinical entity (Table 1).

Furthermore, the practical utility of OCT in photic retinopathy has been extensively evaluated, discussed, and validated [47,48,49,50,51,52,53,54,55,56]. Comander et al. also demonstrated that OCT findings of outer retinal disruption strongly correlated with persistent scotomas in patients with solar retinopathy, allowing for early prognostic counseling [54]. Similarly, Jorge et al. found that the integrity of the ellipsoid zone on OCT strongly correlates with visual outcomes in patients with chronic solar retinopathy [76].

Although steroid therapy showed a quick visual recovery, not all treated patients experienced complete visual recovery and symptom relief, indicating no superiority over conservative care in terms of final visual acuity. Additionally, while treating individuals with photic retinopathy, ocular adverse effects of steroids, such as central serous chorioretinopathy, intraocular pressure increase, or cataract progression, should be considered [72]. Even if there have been a few successful reports of treating photic retinopathy with steroids, prevention is now the best course of action.

## 7. Conclusions

In conclusion, photic retinopathy is a highly avoidable ocular disease, particularly with the use of appropriate protective gear. Suitable patient education is essential, particularly for younger patients whose interest regarding lasers and eclipses might expose them to several cycles of optical radiation.

More research may assist clarify the effectiveness of corticosteroids and/or antioxidants in treating individuals with chronic or one-time UV damage, trying to better understand how these drugs can improve the structural and functional recovery of the damaged retina. Finally, reducing exposure to UV radiation and promoting the use of protective devices (sunglasses, welder’s glass, films, filters, etc.) may be crucial and protective for all individuals, as the precise mechanisms causing macular degeneration have been identified.

## Figures and Tables

**Table 1:** Clinical features, optical coherence tomography diagnosis, and management of different clinical entities of photic retinopathy.

Etiology	Clinical and Diagnostic Features	Optical Coherence Tomography (OCT)	Treatment
**Solar retinopathy**	**Bilateral but asymmetric**, with greater impact on the dominant eye. **Symptoms:** Blurred vision → **scotoma within hours**. A light spot near fixation **before scotoma/metamorphopsia [73]**. **Color vision defects:** Mainly **blue-yellow axis**.	**Acute phase [1]:** Hyperreflective areas → later replaced by hyporeflective cystic spaces (rectangular, receptor loss) [54]; Frequent Verhoeff membrane and ellipsoid line disruption [74]; Severe cases: possible external nuclear layer and external limiting membrane disruption. **Chronic phase [19]:** Broad hypo-reflective area at the photoreceptor interface; “Outer retinal hole” in the fovea → discontinuity in the outer retinal layers; The myoid and ellipsoid zones may exhibit focal disruption; Choroidal abnormalities.	Full vision recovery without special therapy within weeks to six months after exposure [62]; Antioxidants would offer a protective role (Vitamin C) [65]; Methylprednisolone in acute photic retinopathy [67,68,69,72]; No evidence of treatment in chronic cases [72].
**Laser pointer maculopathy**	More severe forms compared with solar lesions [29]. **Yellowish lesions** appear in acute phase, later leading to **pigment changes [1,5]**. Evolution to **cystic lesions** in the external retina, replacing lost photoreceptors [55].	**Acute phase:** Marked hyperreflectivity at the fovea, involving the photoreceptor layer, RPE, outer retina, and ellipsoid zone disruption [62]. **Chronic phase:** Ellipsoid zone loss, focal atrophy of the outer retinal layers, and hyporeflective cavitations within the RPE [3]; Focal rounded pattern → lesions caused by third parties [1]; Retinal streaks → self-inflicted lesions [1].	
**Arc welding maculopathy**	**Acute welding toxicity is** very rare, most common due to **chronic exposure**. **Retinal changes** are frequent but have **minimal impact on vision**.	**Acute phase:** Retinal hyperreflectivity is widespread, especially in the ellipsoid zone and RPE [75]. **Chronic phase:** Gradual outer retinal thinning, ellipsoid zone disruption, and modest foveal atrophy [56]; Outer retinal streak lacks hyperautofluorescence.	
**Iatrogenic photic maculopathy secondary to** **ocular surgery**	Clinical presentation is similar to other photic retinopathies. More extensive lesions, often **above or below the fovea** (due to lack of direct foveal fixation) [1,41].	Lesions frequently below the fovea, increased reflectivity zone affecting all the retinal layers [44]; Changes ranging from subtle ellipsoid zone alterations to pronounced outer retinal atrophy and cystoid spaces in severe cases [41,54].	

## Data Availability

No new data were created or analyzed in this study. All the data are contained within the article.
